# Geranyl-geraniol addition affects potency of bisphosphonates—a comparison in vitro promising a therapeutic approach for bisphosphonate-associated osteonecrosis of the jaw and oral wound healing

**DOI:** 10.1007/s10006-021-00982-8

**Published:** 2021-08-15

**Authors:** Marius Otto, Christine Lux, Tilo Schlittenbauer, Frank Halling, Thomas Ziebart

**Affiliations:** 1 Department for Pediatric Surgery, Pediatric Urology and Pediatric Orthopedics, DRK Children’s Hospital Siegen gGmbH, Wellersbergstrasse 60, 57072 Siegen, Germany; 2 Department for Oral and Maxillofacial Surgery, Interdisciplinary Head & Neck Oncology Laboratory, University Hospital Giessen and Marburg, Campus Marburg, Baldingerstrasse, 35033 Marburg, Germany; 3 Department of Otorhinolaryngology, Section of Oral and Maxillofacial Surgery, University Hospital Augsburg, Stenglinstraße 2, 86156 Augsburg, Germany; 4 Private Practice PD Dr. Dr. Frank Halling, Gesundheitszentrum Fulda, Gerloser Weg 23a, Fulda, Germany; 5 Clinic for Oral and Maxillofacial Surgery, DRK Hospital Alzey, Kreuznacher Str. 7-9, 55232 Alzey, Germany

**Keywords:** Bisphosphonate-associated osteonecrosis of the jaw, Geranyl-geraniol, Cell viability, Migration ability

## Abstract

**Purpose:**

Analysis of the influence of geranyl-geraniol (GG) addition on four bisphosphonate derivatives regarding their influence on cell viability and migration ability of bone metabolism and endothelial cells in vitro.

**Methods:**

Clodronate, pamidronate, ibandronate, and zoledronate were observed with and without GG addition, for their effect on human osteoblasts (HOB), normal human dermal fibroblasts (NHDF), human endothelial progenitor cells (EPC), and endothelial cells of the human umbilical cord (HUVEC) using migration-, MTT-, and colony-forming cell assays.

**Results:**

Data pointed to a depressing effect of all bisphosphonates on the migration ability of NHDF, EPC, and HOB. MTT assay demonstrated a decreased cell viability of HUVEC of all bisphosphonates in a 50 μM concentration and of NHDF when treated with 50 μM of clodronate, ibandronate, or zoledronate. Tested drugs showed a depressing effect on colony-forming potential of EPC even in a 5 μM concentration. GG addition demonstrated an attenuate impact on bisphosphonate effect on all primary cell cultures, respectively.

**Conclusion:**

In vitro comparison showed that the addition of GG weakens the effect of all bisphosphonates examined. It supports investigations that suggest GG to be able to prevent bisphosphonate-associated osteonecrosis of the jaw (BP-ONJ) in vivo. Future clinical trials may discover the local therapeutic use of GG for the prevention of BP-ONJ.

## Introduction

Bisphosphonates are osteoanabolic drugs commonly used in treatment of bone diseases including malignant bone neoplasia, bone metastasis, multiple myeloma, Paget’s disease, and osteoporosis [1, 2]. Despite their therapeutical properties that slow down bone remodeling by inhibiting the bone-degrading osteoclasts, one main side effect is the development of osteonecrosis of the jaw (BP-ONJ) [3, 4]. The first description of BP-ONJ was made by Marx and colleagues in the early 2000s who described patients to have “bony lesions of the upper and lower jaw” [5, 6]. Based on these initial clinical symptoms, further studies have also shown that bisphosphonates have a depressive effect on wound healing of the oral mucosa and the keratinocytes and fibroblasts involved [7, 8]. Bisphosphonates also affect vascularization within wound healing processes by inhibiting this crucial step for tissue regeneration via antiangiogenic effects. In this context, there are inhibitory influences on the proliferation of endothelial cells themselves [9, 10], as well as a reduction of VEGF levels [11]. Numerous studies have already looked for adequate curative therapies for BP-ONJ [12]. The diterpenem geranyl-geraniol (GG), a metabolite in the mevalonate metabolism, plays a central role in this context. It is already known that the inhibitory influence of bisphosphonates on enzymes of the mevalonate metabolism can be reduced by addition of GG. Via inhibition of enzymes in the mevalonate pathway, nitrogenous bisphosphonates lower the level of geranyl-geraniol, which is required for membrane localization of intracellular proteins, especially small GTP-binding proteins and thereby promoting a various number of signaling pathways, e.g., prevention of bone-reabsorption and inhibition of osteoclast formation. A interference of these key pathways reduces cell migration, cell metabolism, and finally leads into apoptosis [13]. The aim of this study was therefore to investigate if different bisphosphonate derivatives influence osteoblasts and wound healing cells like normal human dermal fibroblasts (NHDF) and angiogenic cells [14] in vitro. In particular, the antiangiogenic effect on endothelial progenitor cells (EPC) and human umbilical vein endothelial cells (HUVEC) was analyzed. Another key aspect was the investigation of the effect of GG addition on the possible reduction in the inhibitory effect of bisphosphonates on mevalonate metabolism.

## Material and methods

### Cell cultivation

According to the protocols of the respective manufacturer, the cell cultivation of human osteoblasts (HOB) (no. C-12720, PromoCell, Heidelberg, Germany), HUVEC (no. CC-2517, Lonza, Basel, Switzerland), and NHDF (no. CC 7049, Lonza) was carried out under standardized conditions (SC, 37 °C, 95% humidity, 5% CO_2_) and in suitable cell culture media plus supplements. Cell culture media with the following components were used for the present cell culture experiments. For the incubation of HUVEC Endothelial Cell Growth Basal Medium (500 ml EBM, Lonza Group AG, Basel, Switzerland, plus 10% fetal calf serum, 50 μg/ml gentamicin, 50 ng/ml amphotericin B, 12 μg/ml bovine brain extract, 1 μg/ml hydrocortisone, and 10 ng/ml epidermal growth factor). For NHDF Dulbecco’s Modified Eagle’s Medium (500 ml DMEM, Lonza Group AG, plus 500 ng/ml basic fibroblast growth factor, 1% streptomycin-penicillin-neomycin mixture, and 10% fetal bovine serum). For HOB Dulbecco’s Modified Eagle Medium (500 ml DMEM, Gibco Thermo Fisher Scientific, Waltham, MA, USA, plus 1% L-glutamine, 10% fetal bovine serum, 1% streptomycin-penicillin-neomycin mixture, 20 μg dexamethasone, and 150 mg ascorbic acid). EPC were isolated as described below and cultivated with Endothelial Cell Basal Medium (500 ml EBM; Lonza Group AG, plus 12 μg/ml Bovine Brain Extract, 20% fetal bovine serum, 1 μg/ml hydrocortisone, and 0.5 ml human epithelial growth factor). In the following, the term “suitable nutrient medium” is chosen when speaking of the cell media described here. Cell splitting and reseeding were carried out at a confluence of 90% on the culture bottle bottom. Passages P2–P5 were used for the experiments, while cells which had passed passage 5 were discarded.

For experiments, the culture bottles were removed from the incubator and 5 ml of trypsin was added for an incubation period of 4 min. Subsequently, the trypsin effect was weakened by adding 10 ml of the respective cell medium. The cell enzyme medium solution was distributed evenly on centrifuge tubes and centrifuged for 5 min at 1600* g*. After removing the solution, the cell pellet located at the bottom of the tube was removed. The cells were either resown or used in the experiments after they have been counted. Therefore, a Neubauer counting chamber (Optik Labor, Friedrichsdorf, Germany) and 50 μl trypan blue was used.

### Isolation of EPCs

The EPC were isolated from human peripheral blood by density centrifugation according to a well-established method by Vasa et al. from buffy coats generated at the Transfusion Center of the University Medical Center Mainz [15]. For this purpose, the “peripheral blood” from buffy coats was mixed with a PBS solution (no. D 8537, Sigma-Aldrich, St. Louis, MO, USA) in a ratio of 1:1. In a 50 ml Falcon tube, 15 ml of Ficoll® (sucrose-epichlorohydrin copolymer, no. 10771, Sigma-Aldrich) was applied and carefully overlaid with 25 ml of the prepared Buffy-Blood-PBS mixture. This was followed by centrifugation at 2000* g* for 20 min without a brake. This serves to separate the lymphocyte layer from erythrocytes and granulocytes. Then, the EPCs could be carefully pipetted out of the interphase between Ficoll® and serum layer and transferred again into a 50 ml Falcon tube. In order to obtain an EPC cell pellet, another density centrifugation was carried out at 2000* g* for 10 min with a brake. The cell pellet present at the bottom of the tube could be filled with PBS after carefully removing the excess and fed to a new washing process. This purification process continued until the excess appeared clear. After the last purification step in which the excess was discarded, the cell pellet was filled up to exactly 10 ml and, after gentle mixing, 500 μl cell suspension was removed using a sterile pipette. The cell number was determined in a Neubauer counting chamber and the remaining cells were centrifuged again. The excess of PBS was removed and the cell pellet obtained was fed again to an EPC medium. A concentration of 8 × 106 cells/ml EPC medium was prepared for culture bottles. In order to guarantee, a sufficient adherence of the EPC culture bottles required a fibronectin coating. This was achieved by preparing a fibronectin-PBS mixture in a ratio of 1:100 1 h before sowing. Each 75 cm^2^ culture flask (cat# 658,175, Greiner Bio-One GmbH, Kremsmünster, Austria) was coated with 5 ml fibronectin-PBS mixture and left there for at least 1 h at room temperature. Finally, cells were removed carefully and in each case, 10 ml of the cell-medium mixture was introduced into a 75 cm^2^ culture flask. The incubation was carried out for 3 days in an incubator at SC. Only adherent cells were used for further experiments on day 3 after isolation. The non-adherent cells could be removed from the culture bottle by PBS washing. Twenty-four hours before the start of the experiment, new culture medium was added to the EPC population.

### Migration assay

**Fig. 1:**
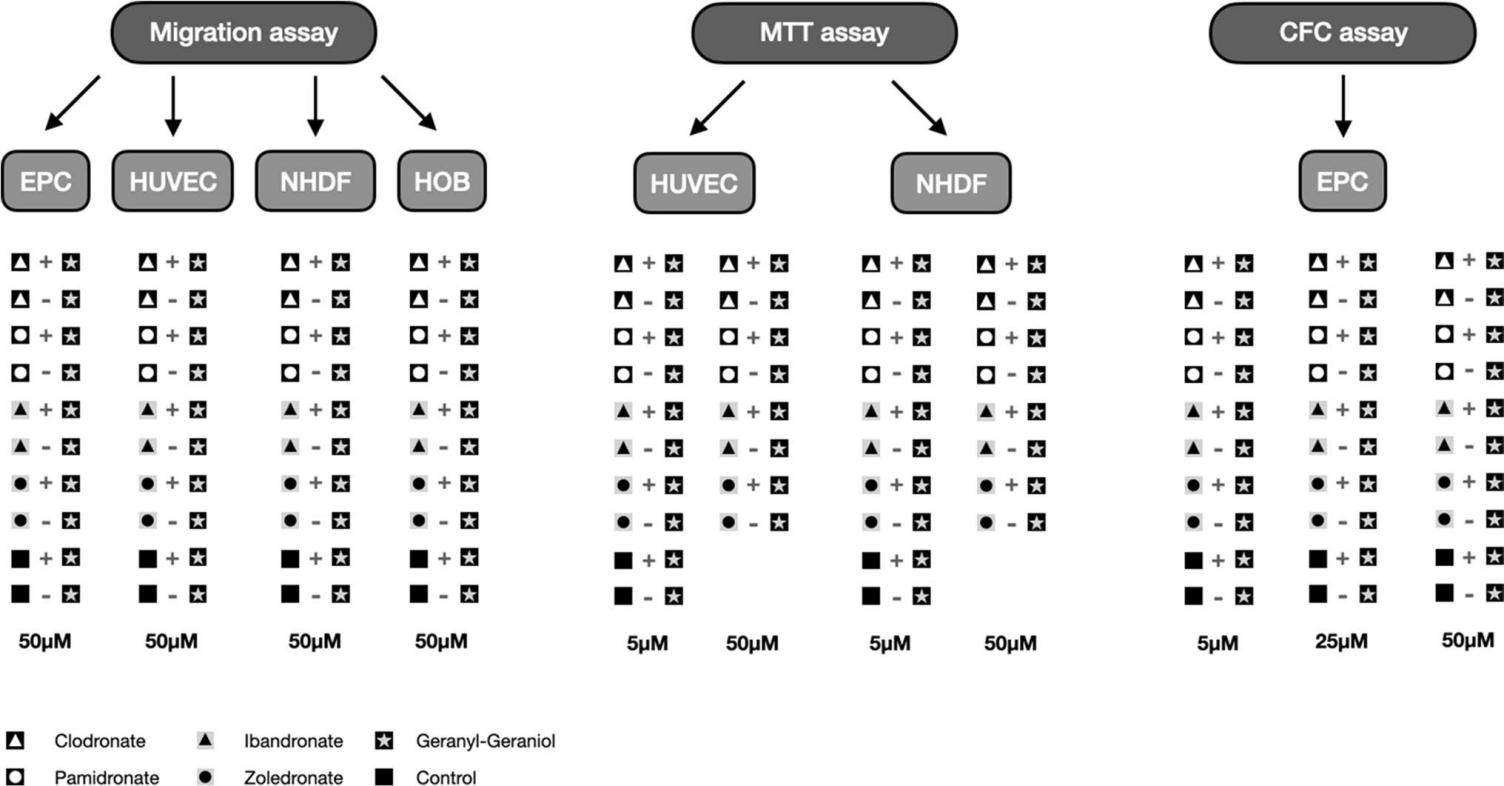
Flowchart of the different in vitro testings; + with,—without

Four different human primary cell cultures (EPC, HUVEC, HOB, NHDF) were cultivated in Boyden migration assays [16, 17] and their migration ability was examined under incubation with the four bisphosphonates to be analyzed and the addition of geranyl-geraniol (Fig. 1).

In short, applied cell cultures were divided into two groups after two (HUVEC, HOB, NHDF) or three (EPC) days. While all culture flasks in group I received an addition with geranyl-geraniol (10 μM), 1.16 μl DMSO per milliliter of medium was added to the culture bottles in group II to compensate for the solvent differences and the possibly associated inaccuracies. In addition, the bisphosphonates clodronate, ibandronate, pamidronate, and zoledronate were added in a concentration of 50 μM. One culture bottle per cell culture from groups I and II remained as a control group without further treatment. The incubation was carried out under SC for 72 h. Finally, the cell medium was removed; the cells were detached by using trypsin and counted again.

Boyden chambers in 24-well format (ThinCertTM, no. 662 638, Greiner Bio-One GmbH) were used for the further experimental approach. The PET membrane used had a pore size of 8 μm. Before the cell suspension solution was introduced, the upper inserts were pretreated with 100 μl fibronectin in a concentration of 10 μg/ml. This had to be carefully eliminated after an hour and initially served for cell adhesion to the bottom of the chamber. While each upper compartment was filled with 400 μl of the specific cell suspension medium, the lower chamber contained 800 μl medium with a selective growth factor. VEGF (2.5 μl/ml medium) was used as the chemotactic active substance in the EPCs and the HUVEC cells, PDGF (2 μl/ml medium) in the HOB and NHDF (2.5 μl/ml medium) in the fibroblasts. The Boyden chamber was capped and incubated for 24 h.

On the next day of the experiment, 600 μl of cell-specific medium with 6 μl of calcein/well (Calcein-AM solution, no. C1359-100UL, Sigma-Aldrich) was applied to a transparent 24-well plate. After carefully removing the cell medium from the incubated Boyden chambers, the inserts were immersed in a calcein bath for about 1 h and then carefully cleaned in a sterile PBS solution. A black 24-well plate with 500 μl trypsin/well was prepared. In there, the upper Boyden chamber inserts are inserted and incubated for 5 min. The migrated cells were detached from underneath of the membrane by carefully tapping and shaking the well plate. In order to be able to quantify the migration, after thorough mixing of the individual cell solutions, these were transferred to a well plate with a transparent bottom. The photometric evaluation took place with an emission of 538 nm with an excitation of 485 nm wavelength and 100 ms/well.

### MTT assay

The MTT assay using 3-(4,5-dimethylthiazol-2-yl)-2,5-diphenyltetrazolium bromide was first described by Mosmann in 1983 [18]. The assay is a way of quantitatively determining the number of living cells by photometric measurements and is therefore a measure of cell viability. After forced lysis of the treated cells and the solution of the crystals by solubilization, a colorimetric analysis is carried out in a photospectrometer at a wavelength of 550 nm. This test approach was carried out on the HUVEC and NHDF cells (Fig. 1). The cell batches cultivated could be used here. After detaching the cells from the culture bottle, the number of cells was determined and the cells were transferred to the prepared 6 well plates. For each well, 1 × 10^5^ cells were placed in their suitable nutrient medium (2.5 ml/well) and incubated for adherence for 24 h under standard conditions. The next day, after changing the medium, the 6-well plates could be divided into 2 groups. In the first group, geranyl-geraniol (1.16 μl/ml medium) was applied. In the second group, DMSO was added in the same amount to compensate for the solvent differences.

In addition, with the exception of the control groups, the bisphosphonates clodronate, ibandronate, pamidronate, and zoledronate were added to the test batches in the concentrations 5 μM and 50 μM. The incubation was then continued in the incubator for a further 72 h. On the 4th day after transferring the cells into the 6-well plates, the MTT assay was prepared so that the viability of the cells could then be represented photometrically. For this purpose, an MTT stock solution could be added to the cell test series (250 μl/well). After 4 h of incubation, this was carefully removed from the well plates together with the medium. The well was then washed out in two successive steps using PBS.

In the following step, a lysis buffer consisting of 49 ml propanol and 1 ml 2 M HCl could be prepared. This served to detach the remaining adherent cells and was added to the well plates, which could then be incubated for 25 min.

After the mixing, 1000 μl of the buffer cell mixture was transferred into the prepared MTT measuring tubes using a pipette. In order to determine the blank value, a measuring tube was only equipped with lysis buffer. The remaining measuring tubes could be evaluated in a photometer at a wavelength of 550 nm (LAB-Systems, Hagedorn, Germany).

### Colony-forming cell assay

The colony-forming cell (CFC) is an in vitro assay that is used in research with haematopoetic stem cells. Based on their ability to proliferate, differentiate, and form colonies in the appropriate cell medium under stimulation with cytokines. This latter property can be quantified using the CFC assay. Therefore, the non-adherent EPCs were washed out 3 days after incubation. The adherent cells were removed from the culture flask using trypsin, centrifuged, and counted according to the standards described. The target cells were applied in a concentration of 3 × 10^5^ cells per 400 μl EPC medium. Four milliliter portions of a medium containing methyl cellulose, the so-called Methocult (Stemcells Technologies, Cologne, Germany), were filled in 15 ml tubes and 4 μl VEGF was added. The EPC suspension was applied to each of the 4 ml Methocult-VEGF tubes. Each tube contained 400 μl EPC cell medium with approximately 300,000 EPCs.

Finally, the tested bisphosphonates (clodronate, ibandronate, pamidronate, and zoledronate) were concentrated at 0; 5, 25, and 50 µM added and vortexed. In order to investigate the effect of geranyl-geraniol, the batches were divided into two groups. Group I received an additional 1.16 μl/ml geranyl-geraniol in each tube. Group II was applied in equal amounts to compensate for the solvent differences. The tubes were incubated for 5 min to ensure the escape of air bubbles after the mixing process. In addition, two control groups without bisphosphonates but with geranyl-geraniol or in an analogous amount DSMO were set up. Subsequently, each group (with/without GG) and per bisphosphonate in each individual concentration could be equipped with a 10 ml plate with three 35 ml well plates and labeled for recognition (Fig. 1). The bubble-free Methocult-VEGF-bisphosphonate cell mixture with and without GG was applied. A cell concentration of 10^5^ cells was applied to each of the 35 ml well plates and distributed evenly. The 10 ml plate could be wetted with 2 ml PBS to ensure an even supply of moisture. This was followed by a 10-day incubation again under standard conditions. After 10 days, the colonies formed were counted under a light microscope.

### Statistics

All experiments were carried out once, evaluated by two specialists in oral and maxillofacial surgery and the respective results presented here. Resulting data of this work are presented as mean and standard deviation. The statistical analysis was carried out using an ANOVA analysis of variance and a post hoc *t*-test. A Bonferroni-Holm corrected *p* < 0.05 indicates significance within graphical evaluation.

## Results

### Bisphosphonates reduce migration ability of bone metabolism cells

The migration assay showed that bisphosphonates influence the migration behavior of HOB at a concentration of 50 μM. Comparing the control group without GG addition and the respective test series with bisphosphonate addition only, a generalized significant inhibition of the migration behavior of human osteoblasts was found.

The inhibitory effect on migration was most pronounced with zoledronate. The migration was reduced on average by a little more than half compared to the control (45%, *p* < 0.05, Bonferroni-Holm correction, Fig. 1). Clodronate showed the least inhibition on migration behavior in this comparison. With the addition of GG to the respective bisphosphonate batches, it was only possible to demonstrate a significant inhibition of the ability to migrate in the pamidronate and zoledronate groups compared to the control group without GG addition. Clodronate and ibandronate did not significantly influence the migration behavior of the HOB compared to the control group when GG was added (Fig. 1).

**Fig. 2:**
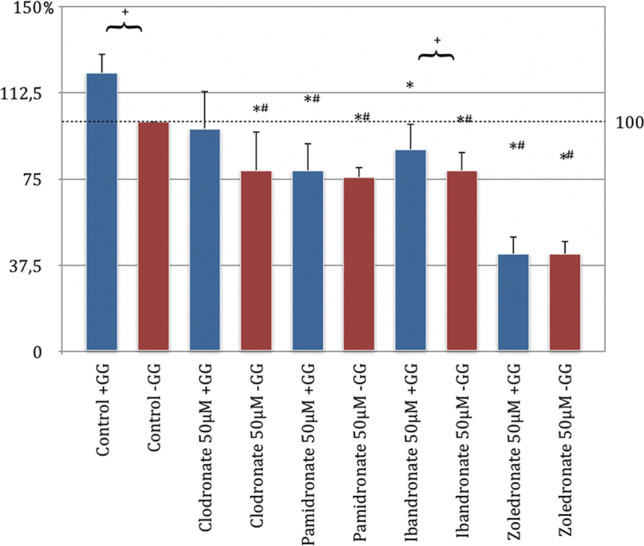
Migration assay of human osteoblasts (HOB) with substituted bisphosphonates in 50 μM concentration; # significantly enhanced migration compared to control w/o geranyl-geraniol; * significantly enhanced migration to control w geranyl-geraniol; + significantly enhanced migration w geranyl-geraniol compared to the same bisphosphonate w/o geranyl-geraniol addition. *p* < 0.05 indicates statistical significance

When evaluating the influence of the ability to migrate by adding geranyl-geraniol to the respective groups, a generalized increase in the tendency to migrate was found. However, this increase was only significant in the control and ibandronate groups. In summary, it was found that after the application of GG, the ability to migrate was increased compared to the same test series without GG addition (Fig. 2).

When evaluating the results of the behavior of the bisphosphonates on NHDF, it could be shown that all bisphosphonates used without GG addition showed a significant inhibition of migration compared to the control group without GG (*p* < 0.05, Bonferroni-Holm correction, Fig. 2). The NHDF was also most strongly inhibited by zoledronate, followed by ibandronate and pamidronate. The clodronate had the weakest negative effect on the migration behavior of the cells (Fig. 2).

**Fig. 3:**
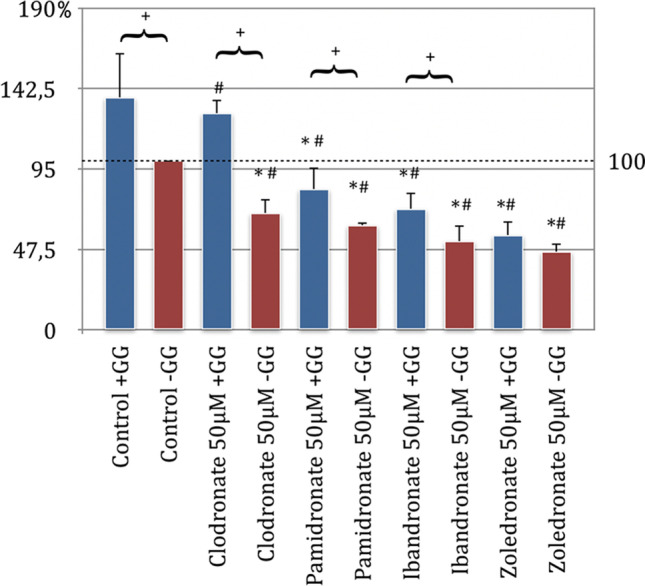
Migration assay of fibroblasts (NHDF) with substituted bisphosphonates in 50 μM concentration; # significantly enhanced migration compared to control w/o geranyl-geraniol; * significantly enhanced migration to control w geranyl-geraniol; + significantly enhanced migration w geranyl-geraniol compared to the same bisphosphonate w/o geranyl-geraniol addition. *p* < 0.05 indicates statistical significance

Pamidronate, ibandronate, and zoledronate, each with geranyl-geraniol addition, showed a significant inhibition of cell migration compared to the control group without added GG. Meanwhile, clodronate under the influence of GG showed an increase in migration compared to the same control group, which turned out to be insignificant. When analyzing the migration profile, a significant increase in the migration behavior could be determined by adding geranyl-geraniol in the clodronate, pamidronate, ibandronate, and control groups. This was not the case with zoledronate (Fig. 3).

**Fig. 4:**
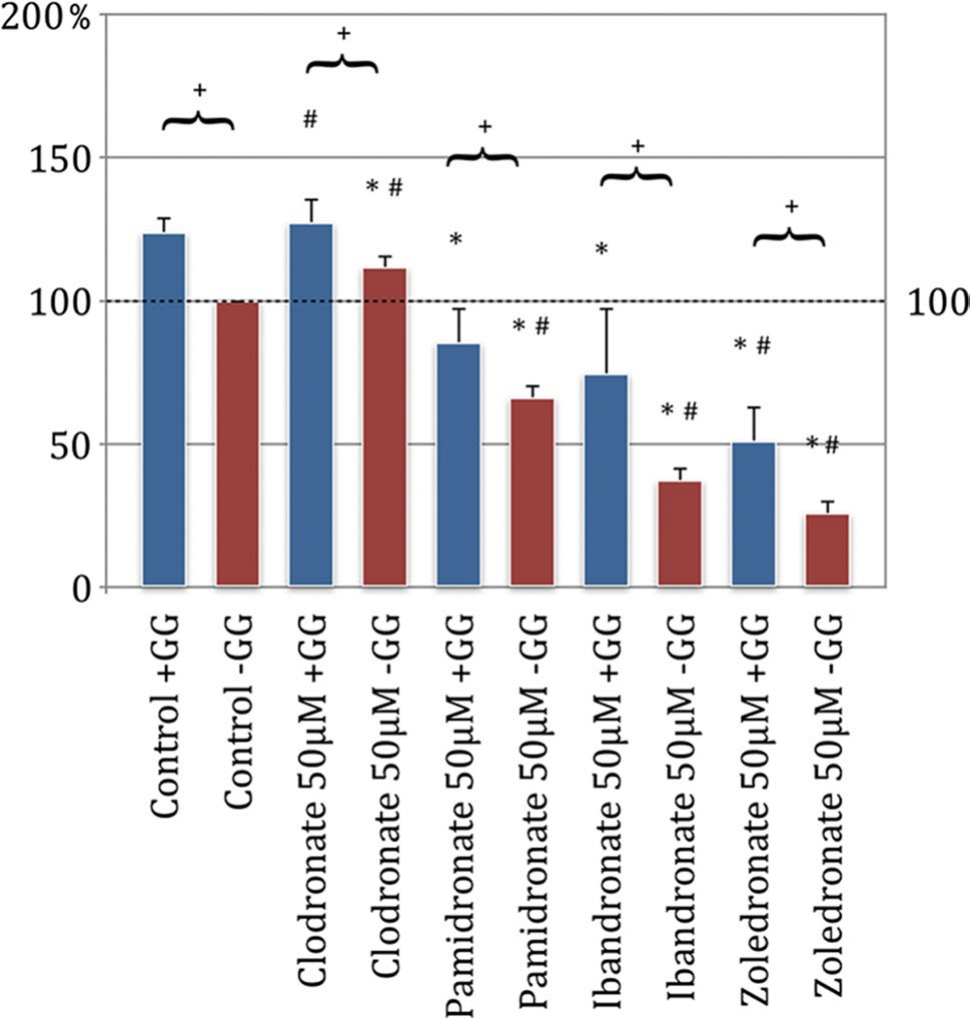
Migration assay of human umbilical cord endothelial cells (HUVEC) with substituted bisphosphonates in 50 μM concentration; # significantly enhanced migration compared to control w/o geranyl-geraniol; * significantly enhanced migration to control w geranyl-geraniol; + significantly enhanced migration w geranyl-geraniol compared to the same bisphosphonate w/o geranyl-geraniol addition. *p* < 0.05 indicates statistical significance

Following the knowledge already presented, the bisphosphonates ibandronate, pamidronate, and zoledronate significantly inhibit the migration behavior of the HUVEC cells as well. Zoledronate showed the strongest inhibition, followed by ibandronate and pamidronate (*p* < 0.05, Bonferroni-Holm correction, Fig. 4).

Clodronate incubation presented no inhibitory effect at all. When assessing the cells under clodronate application with geranyl-geraniol, a significant increase in migration was observed compared to the control group without GG. For the cells with GG treated with ibandronate and pamidronate in comparison to the control group without GG, the following can be determined: the significant inhibition of the migration behavior, which can be observed under the influence of pure bisphosphonate, was eliminated by the additional incubation of the cells with geranyl-geraniol.

Only the influence of zoledronate with GG reduced migration ability significantly compared to the control group without GG. In comparison of the groups with and without GG, all five samples showed that GG significantly weakens the inhibitory effect on the migration behavior of HUVEC. Consequently, the addition of GG leads to a significant increase in the migration behavior of the HUVEC cells in all tested groups (Fig. 4).

**Fig. 5:**
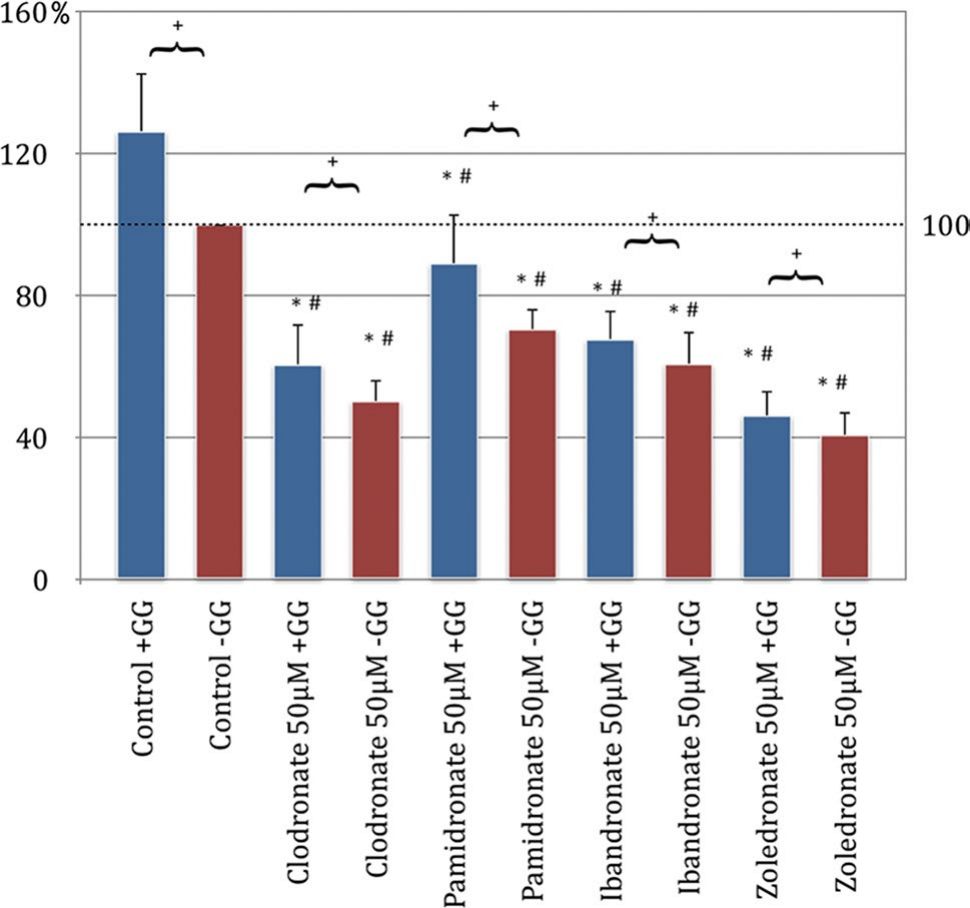
Migration assay of endothelial progenitor cells (EPC) with substituted bisphosphonates in 50 μM concentration; # significantly enhanced migration compared to control w/o geranyl-geraniol; * significantly enhanced migration to control w geranyl-geraniol; + significantly enhanced migration w geranyl-geraniol compared to the same bisphosphonate w/o geranyl-geraniol addition. *p* < 0.05 indicates statistical significance

All bisphosphonates in their tested concentration without GG addition showed a significant inhibition in their migration behavior compared to the control group without GG on EPC (*p* < 0.05, Bonferroni-Holm correction, Fig. 4). Zoledronate was shown to be the strongest inhibitor, followed by clodronate. The weakest inhibitory effect on the EPCs was demonstrated by pamidronate (Fig. 5).

The comparison of the control group with GG addition and the experimental approaches of bisphosphonates with geranyl-geraniol also led to significant changes in migration. Each of the bisphosphonates tested with GG application reduced the EPC’s desire to migrate compared to the control group with geranyl-geraniol (Fig. 5).

The group analysis showed that the addition of GG to each primary cell culture compared to an incubation without geranyl-geraniol had a significant positive influence on migration ability of the cells in all test approaches.

### Bisphosphonates inhibit colony forming of EPC

**Fig. 6:**
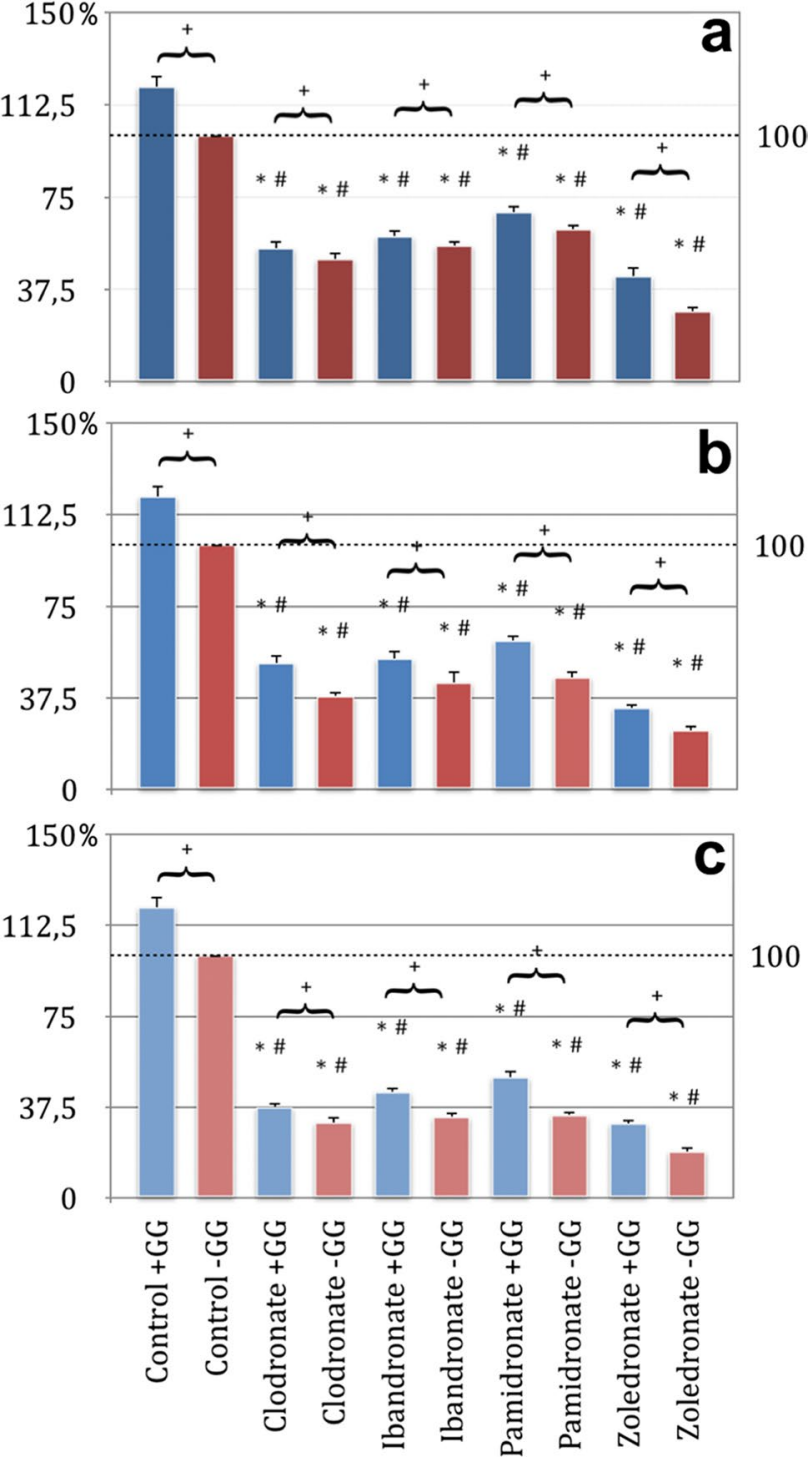
Colony-forming unit assay of endothelial progenitor cells (EPC); (a) 5 μM concentration of substituted bisphosphonates; (b) 25 μM concentration of substituted bisphosphonates, (c) 50 μM concentration of substituted bisphosphonates; # significantly enhanced CFU compared to control w/o geranyl-geraniol; * significantly enhanced CFU compared to control w geranyl-geraniol; + significantly enhanced CFU w geranyl-geraniol compared to the same bisphosphonate w/o geranyl-geraniol addition. *p* < 0.05 indicates statistical significance

In the experimental setup of the colony-forming assay, the aim of EPC to form a colony in their suitable nutrient medium should be investigated by incubation with the bisphosphonates clodronate, ibandronate, pamidronate, and zoledronate. The results showed that in the bisphosphonate test series without GG addition, there was a significant concentration-dependent reduction in colony formation behavior compared to the control group without GG application (*p* < 0.05, Bonferroni-Holm correction, Fig. 6).

This reduction in the number of colonies was most evident at a high bisphosphonate concentration (Fig. 6c) and the smallest at a 5 μm concentration (Fig. 5a). In addition, the effort to form colonies was most significantly reduced in the highly potent nitrogen-containing zoledronate, followed by the clodronate and ibandronate test series. Most of the colonies formed arose under pamidronate addition (Fig. 6a–c).

When considering the test series, which were additionally treated with GG in addition to the bisphosphonate, a concentration-dependent reduction in the number of colonies was found compared to the control group without GG addition. However, the inhibitory tendency was not as pronounced as for the bisphosphonate groups without GG influence.

The results indicate a connection because the higher the bisphosphonate concentration, the more the number of colonies is reduced. The application of GG to the bisphosphonate test series resulted in a significant increase in colony formation behavior in the EPC compared to the test series without the addition of GG.

### Bone metabolism cell viability descends under treatment with bisphosphonates

**Fig. 7:**
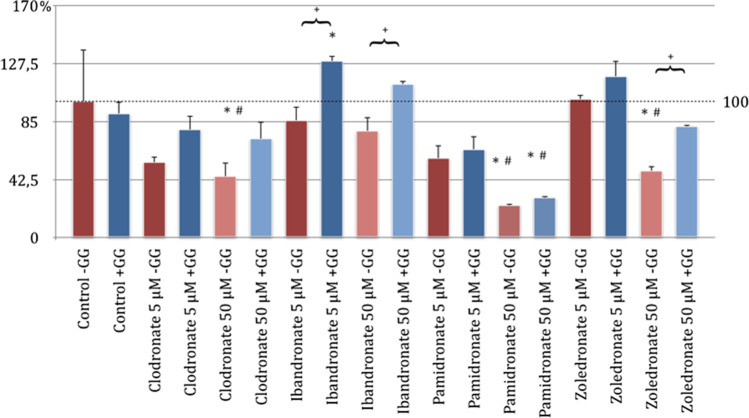
MTT assay of fibroblasts (NHDF) with substituted bisphosphonates in 5 μM and 50 μM concentrations; # significantly enhanced cell viability compared to control w/o geranyl-geraniol; * significantly enhanced cell viability to control w geranyl-geraniol; + significantly enhanced cell viability w geranyl-geraniol compared to the same bisphosphonate w/o geranyl-geraniol addition. *p* < 0.05 indicates statistical significance

**Fig. 8:**
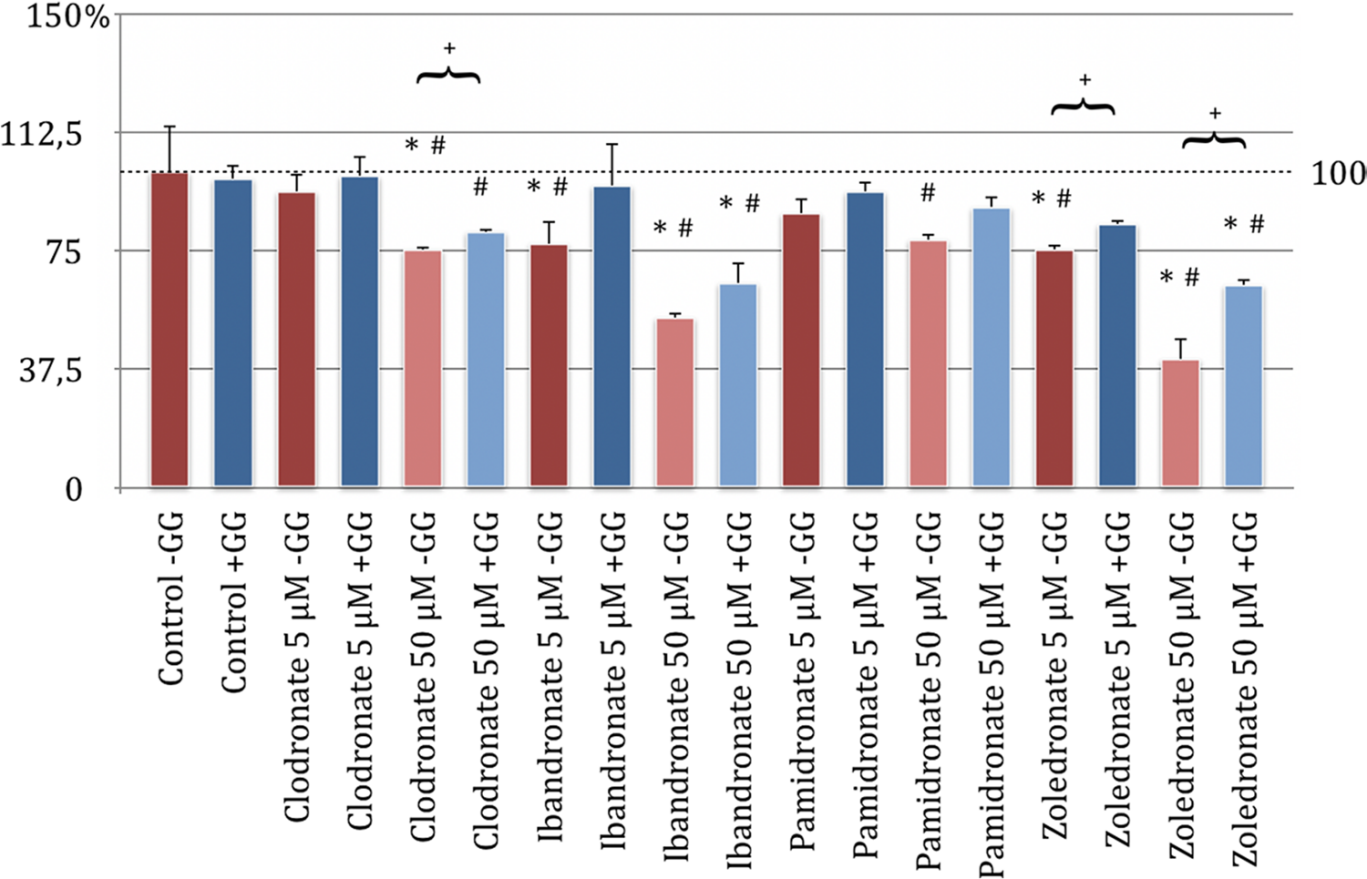
MTT assay of human umbilical cord vein endothelial cells (HUVEC) with substituted bisphosphonates in 5 μM and 50 μM concentrations; # significantly enhanced cell viability compared to control w/o geranyl-geraniol; * significantly enhanced cell viability to control w geranyl-geraniol; + significantly enhanced cell viability w geranyl-geraniol compared to the same bisphosphonate w/o geranyl-geraniol addition. *p* < 0.05 indicates statistical significance

The influence of the bisphosphonates clodronate, ibandronate, pamidronate, and zoledronate in the concentrations of 5 μM and 50 μM on the primary cell lines of the fibroblasts (NHDF, Fig. 7) and the human umbilical venous endothelial cells (HUVEC, Fig. 8) was tested as part of the applied MTT assay. In addition to the effect of the bisphosphonates on the viability of these cells, the influence of geranyl-geraniol was also examined (*p* < 0.05, Bonferroni-Holm correction, Figs. 7 and 8). The comparison between the control group and the bisphosphonates tested without GG addition showed a significant inhibition of the viability of the fibroblasts with clodronate 50 μm, pamidronate 50 μM, and zoledronate 50 μM. Incubation of the cells with ibandronate 50 μM showed no significant effects compared to the control group. The strongest negative influence was demonstrated by pamidronate in this analysis, followed by clodronate and zoledronate (Fig. 7).

The cultivation of the cells with only bisphosphonate administration in the lower concentration of 5 μM led to the findings that clodronate, ibandronate, and pamidronate showed a decrease in viability (the values could not be classified as significant compared to the control without GG addition). In addition, zoledronate had no inhibitory effect (Fig. 7). If the results of the test series, which were substituted for bisphosphonates in the concentrations 5 μM and 50 μM and also with geranyl-geraniol, were compared with the control group without GG addition, only for pamidronate 50 μM was there a significant reduction in the viability of the NHDF (Fig. 7). In the ibandronate test series of the concentrations 5 μM and 50 μM and in the cell set which was incubated with zoledronate 5 μM, the addition of geranyl-geraniol led to a significant increase in viability.

In the remaining series of bisphosphonates, the addition of GG tended to show a reduction in the inhibitory influence compared to the application of bisphosphonate alone. However, this was not significant.

In summary, the group analysis showed that adding GG to the bisphosphonate cell series has a significantly positive effect on cell viability compared to incubation without geranyl-geraniol with ibandronate 5 μM and 50 μM and with zoledronate 50 μM and inhibits the inhibitory effect of bisphosphonate administration.

A concentration-dependent inhibitory effect was demonstrated in all four bisphosphonates tested within MTT assay using HUVEC as seen in Fig. 8. Compared to the 5 μM concentration, the bisphosphonates also showed a greater influence on the HUVEC cells at a 50 μM concentration.

The results of the MTT assay showed that all four bisphosphonates in a 50 μM concentration without GG addition caused a significant inhibition of cell viability compared to the control group. This inhibition was most pronounced with zoledronate, followed by ibandronate and clodronate. The weakest influence compared to the control group without GG addition was recorded with pamidronate (Fig. 8).

In the analysis of the bisphosphonate test series in 5 μM concentration without GG addition and the control group without geranyl-geraniol application, only ibandronate and zoledronate showed a significant inhibition of viability. This was slightly higher for zoledronate than for ibandronate. HUVEC under pure influence of clodronate and pamidronate in a concentration of 5 μM show a tendency to inhibit compared to the control group, but without any significance.

In a comparison of the test series between bisphosphonates with GG addition and the control group without GG addition, the bisphosphonates clodronate, ibandronate, and zoledronate in a concentration of 50 μM showed a significant reduction in cell viability (Fig. 8).

In all groups treated with bisphosphonates, the inhibitory tendency of the drugs could be weakened by the influence of the GG added. A significant weakening of the inhibitory influence was only observed with the application of clodronate 50 μm, zoledronate 5 μM, and zoledronate 50 μM (Figs. 7 and 8).

### The inhibitory effect of geranyl-geraniol on the mechanism of action of bisphosphonates

The migration ability of cells of angiogenesis (EPC and HUVEC) was significantly elevated with geranyl-geraniol addition of all bisphosphonates. Geranyl-geraniol addition led to a significantly improved migration ability of fibroblasts when added to the bisphosphonates clodronate, pamidronate, and ibandronate. Also, a significant increase of migration after geranyl-geraniol addition could be observed in osteoblasts when pretreated with ibandronate. The tests on the viability showed an improvement after geranyl-geraniol addition. The viability of fibroblasts could be significantly increased after geranyl-geraniol addition when pretreatment with ibandronate in 50 μM or 5 μM and zoledronate 50 μM concentration had been performed. In HUVEC, a significantly enhanced viability could be observed after geranyl-geraniol addition in the 50 μM clodronate and 5 and 50 μM zoledronate group. The impact on the typical characteristic of EPC which is the colony-forming property could be significantly improved after geranyl-geraniol addition within all four bisphosphonate groups regardless of the tested concentrations (5, 25, and 50 μM, *p* < 0.05, Bonferroni-Holm correction, Figs. 2, 3, 4, 5, 6, 7, and 8).

## Discussion

Bisphosphonates belong to the category of osteoanabolic pharmaceuticals, used for indications such as osteoporosis, inhibition of the progression of osteolytic metastases, or primary osseous cancer. The therapeutic successes are about their inhibition of the osteoclasts, which is regarded as the key cell for the osteoanabolic effect. The result of reduced bone loss causes a change in bone homeostasis into the direction of bone build-up [19, 20]. However, bisphosphonates do not act exclusively on the osteoclasts. It has been shown that bisphosphonates also influence osteocyte and osteoblast cell lines. The effect of bisphosphonates on osteoblasts was first demonstrated by Khokher and Dandona in 1989, who showed that the addition of bisphosphonates inhibits the proliferation of osteoblasts and their secretion of alkaline phosphatase [21]. This inhibitory influence, which was confirmed later on [22–24], was also observed in the present study. In fact, the migration assay showed a significant inhibition of the migration ability of HOB in all four bisphosphonate preparations, with the strongest effect seen for zoledronate. On average, a relative inhibition of 45% of the ability to migrate in comparison with the control group was recorded.

The effect of bisphosphonates on NHDF is also described in the literature. A cytotoxic effect could be demonstrated and apoptosis-inducing and proliferation-inhibiting properties were seen [25].

Examining the behavior of the most potent bisphosphonate zoledronate on fibroblasts, a cytotoxic effect was found.

Basso and colleagues showed a significantly reduced viability of the fibroblasts at concentrations of 5 μM examining the behavior of the most potent bisphosphonate zoledronate [26]. Similar tendencies can also be seen here, since, e.g., a significant inhibition of NHDF was found in the migration assay carried out with 50 μM bisphosphonate concentration. The inhibitory effect was linear to the relative biological potency of the bisphosphonates tested. Accordingly, zoledronate showed the strongest and clodronate the weakest inhibition of the migration ability. MTT assay also showed a significant reduction in the viability of NHDF for the bisphosphonates clodronate, pamidronate, and zoledronate in a concentration of 50 μM. Only ibandronate administration with 50 μM showed a negative influence. However, this was not significant compared to the control group. In contrast to the study by Basso and colleagues, no significant reduction in viability in the MTT assay was demonstrated for bisphosphonate incubation at 5 μM concentration [26]. The inhibitory effect to be determined did not correlate with their biological potency. As a result, bisphosphonates in lower concentrations do not appear to have a proportional influence on the viability of NHDF.

Furthermore, there is an antiangiogenic effect of bisphosphonates seen within cancer studies. For example, there was a decrease of circulating proangiogenic factors [11], an inhibition of proliferation, adhesion, and migration of endothelial cells [27], and a reduction of circulating endothelial progenitor cells in the blood of patients with BP-ONJ [28] after bisphosphonate medication. In a comparison of the antiangiogenic effects between non-nitrogen and nitrogen-containing bisphosphonates, an inhibition of angio- and vasculogenesis was demonstrated for both substances. The inbounding properties of the nitrogen-containing bisphosphonates zoledronate and pamidronate were most pronounced on HUVEC and EPCs [29]. The presented data of this study also showed an inhibition of bisphosphonates on cells of the vascular system. EPC and HUVEC showed the clear tendency that their migration behavior could be reduced linearly to their biological potency by the addition of the aminobisphosphonates pamidronate, ibandronate, and zoledronate. The non-nitrogen-containing clodronate differed in its effect on the two primary cell cultures tested. Only EPC were inhibited to migrate. MTT assay discovered a dose-dependent effect so that at a concentration of 50 μM, all four bisphosphonates had a negative influence on the viability of the angiogenic cells. Analogously, a generalized inhibition of colony formation behavior by aminobisphosphonates was demonstrated for EPC. This could be depicted depending on the concentration and linearly with the biological potency of the respective nitrogenous bisphosphonate. As was already evident during the migration of the EPCs, the influence of clodronate in relation to its biological effectiveness is more pronounced than assumed and showed the second-largest concentration-dependent inhibition of colony formation after zoledronate. These results are in agreement with the antiangiogenic effect of clodronate described by Ribatti and Fournier [9, 30]. The follow-up work using the same bisphosphonate preparations within a 3D Matrigel assay in vivo showed a decrease in microvascular density and space after bisphosphonate treatment [7].

Numerous studies propose to assume a multifactorial genesis of BP-ONJ because the exact pathophysiological cause has not been conclusively determined yet [12, 19, 31]. There is agreement that bisphosphonates have a sensitive influence on the homeostasis of the oral cavity and induce the development of BP-ONJ, probably triggered by the mechanism of action of the inhibition of the mevalonate metabolism [2, 19]. A hypothetical therapy option is the substitution of the missing substrates out of that pathway: farnesyl and geranyl–geranyl pyrophosphate. For external substitution, penetration into the corresponding cells is necessary. Here, however, the hydrophilic pyrophosphate portions would decrease the intracellular uptake. Geranyl-geraniol offers an alternative because it is diphosphorylized after penetration through the cell membrane and can then anaploretically maintain the function of the mevalonate metabolism with subsequent post-translational geranylization of important cellular signaling proteins [32]. That is why the influence of GG was examined here. Following the assumption, all primary cell lines incubated with bisphosphonate (EPC, HOB, HUVEC, NHDF) benefited from the addition of GG. An analysis of the influence behavior of GG on the angiogenic cells EPC and HUVEC showed that the migration behavior can be significantly increased by GG addition for all bisphosphonates.

With regard to the viability of the cells, the MTT assay from HUVEC showed that only zoledronate and clodronate at a concentration of 50 μM could be significantly improved by an additional incubation of geranyl-geraniol. Analogous to the migration test, a significant increase in the colony-forming properties of the EPCs was also achieved in the colony-forming assay by adding GG. A positive influence was also observed in the cells of the supporting tissue (NHDF) by adding GG. Thus, an addition of GG resulted in a cytoprotective effect in all test series, which agrees with the results in the literature [13, 33]. In addition to the cytoprotective effect, angiogenic properties of GG are also described, which works via a bisphosphonate-induced dysregulation of specific angiogenic genes [34]. On the one hand, this finding underlines the assumption that the inhibitory influence of the bisphosphonates is triggered by the change through GG in the mevalonate metabolism. On the other hand, it offers an outlook for a possible therapeutic potential, as discussed in the article by Zafer and colleagues [35].

An important limitation of this study is the diversity of the examined cell cultures, which only allow rough interpretations of the bisphosphonate effect. Due to the study design with partly different cell culture-specific materials, the influence of the bisphosphonates on different cell cultures should be investigated. The study thus focuses on the bisphosphonate effect on different cell cultures and their inhibition by the presence of GG. It is therefore important to emphasize that a qualitative analysis per cell type was not the aim of the experiments.

A precise cell-specific investigation of the bisphophonate and GG effect should take place in the following experiments, per primary cell culture. This would also enable a dose–response analysis and, based on the data presented, remains a promising approach for future in vitro studies. Furthermore, this could provide detailed knowledge of whether GG inhibits the respective bisphosphonates to different degrees. For this, only rough correlations and strong trends could be demonstrated here.

A therapeutic solution for the treatment of BP-ONJ is still challenging, but the results of this study as well as further works show to some extent the potential of GG [13, 35]. Topical application would be important in order to avoid a generalized weakening of the bisphosphonate effect, as it could be demonstrated in vitro [13]. Otherwise, it might have a disadvantageous effect, especially in the case of oncological diseases. As part of a possible reevaluation, this potential of GG should be further investigated. Therefore, the topical application is suggested for further experimental settings.

Following this assumption, it is conceivable to use mouth rinses containing GG or to introduce GG into the affected jaw region as part of a surgical intervention in patients with BP-ONJ. It remains to be seen to what extent the in vitro results can be transferred to in vivo studies [13].This therapeutic approach works directly within the pharmacological mechanism of action and would thus offer the possibility of reducing the toxic effects of bisphosphonate therapy locally.

## Conclusion

In conclusion, a generalized inhibitory effect of all bisphosphonates on all primary cell cultures examined could be determined. The migration behavior of HOB, EPC, HUVEC, and NHDF was significantly inhibited by all tested bisphosphonates.

Bisphosphonates have an inhibitory effect on the migration ability of bone metabolism and endothelial cells. Furthermore, they highly influence their cell viability in vitro. Both pharmaceutical properties can be significantly weakened by the addition of GG. As a bridge to therapy in humans, topical application could be important in order to avoid a generalized weakening of the bisphosphonate effect and thus offer the possibility of local reduction of the toxic consequences of bisphosphonate therapy within oral wound healing in general and, above all, BP-ONJ. Furthermore, these findings might be a promising approach for future testings in vivo to gain more information in context of local drugs against BP-ONJ.
